# Design and Preparation of Carbon Based Composite Phase Change Material for Energy Piles

**DOI:** 10.3390/ma10040391

**Published:** 2017-04-07

**Authors:** Haibin Yang, Shazim Ali Memon, Xiaohua Bao, Hongzhi Cui, Dongxu Li

**Affiliations:** 1 College of Civil Engineering, Shenzhen University, Shenzhen 518060, China; 2150150417@email.szu.edu.cn (H.Y.); h.z.cui@szu.edu.cn (H.C.); dongxuli@njtech.edu.cn (D.L.); 2 Department of Civil Engineering, School of Engineering, Nazarbayev University, Astana 010000, Kazakhstan; shazim.memon@nu.edu.kz

**Keywords:** composite phase change materials, expanded graphite, graphite nanoplatelet, energy storage, energy piles

## Abstract

Energy piles—A fairly new renewable energy concept—Use a ground heat exchanger (GHE) in the foundation piles to supply heating and cooling loads to the supported building. Applying phase change materials (PCMs) to piles can help in maintaining a stable temperature within the piles and can then influence the axial load acting on the piles. In this study, two kinds of carbon-based composite PCMs (expanded graphite-based PCM and graphite nanoplatelet-based PCM) were prepared by vacuum impregnation for potential application in energy piles. Thereafter, a systematic study was performed and different characterization tests were carried out on two composite PCMs. The composite PCMs retained up to 93.1% of paraffin and were chemically compatible, thermally stable and reliable. The latent heat of the composite PCM was up to 152.8 J/g while the compressive strength of cement paste containing 10 wt % GNP-PCM was found to be 37 MPa. Hence, the developed composite PCM has potential for thermal energy storage applications.

## 1. Introduction

**Figure 1:**
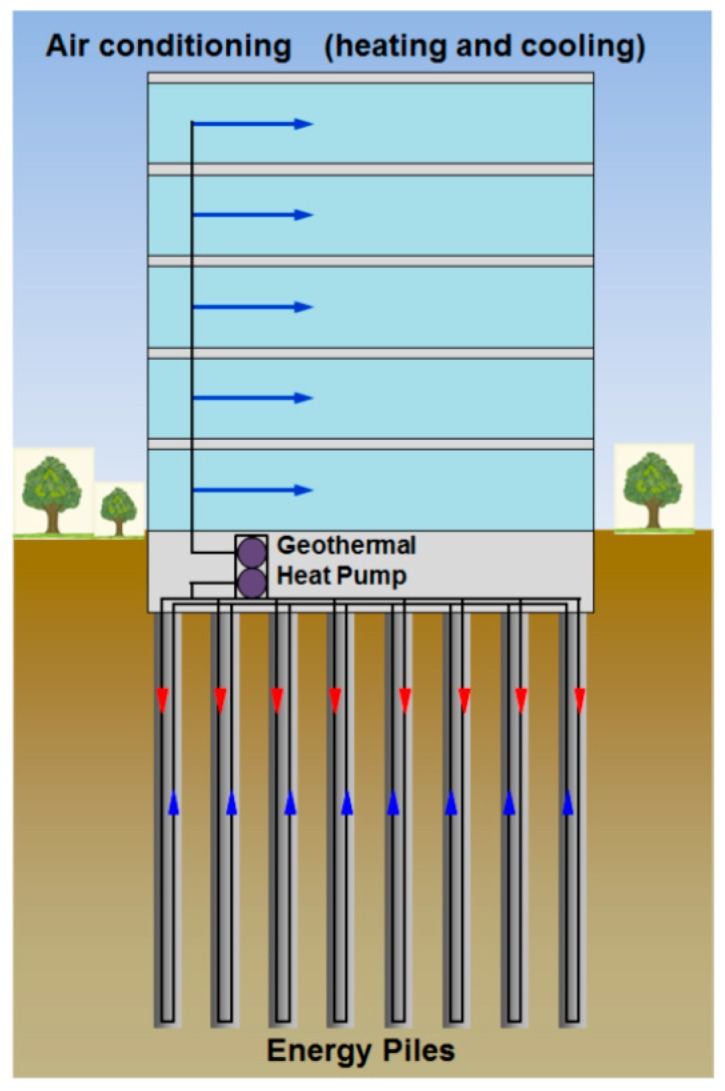
Schematic drawing of energy piles application in building energy efficiency [6].

**Figure 2:**
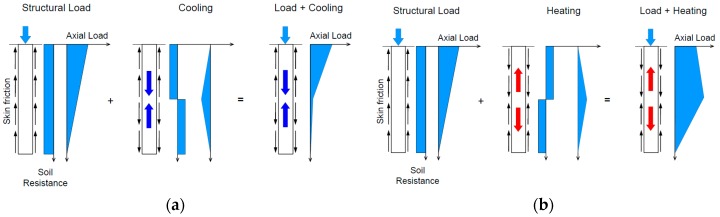
Effect of thermal cycles of the energy piles system on pile stresses [6]. (**a**) Ground cooling reduces stresses along the cross section of the piles which can cause tensile stresses in the piles; (**b**) Heating can cause increased stresses along the cross section of the piles.

The rapid increase in global energy consumption has led to serious issues such as depletion of fossil fuels and degradation of the environment [1,2]. According to statistics, the world’s energy consumption will grow by 48% between 2012 and 2040 [3]. Hence, energy policy makers and researchers are paying a lot of attention to the building sector as it is responsible for around 30% of the total global energy consumption [4,5]. Energy piles—A fairly new renewable energy technique—use a ground heat exchanger (GHE) in the foundation piles to supply heating and cooling loads to the supported building. Figure 1 is a schematic drawing of energy piles application in building energy efficiency. Geothermal energy can sustainably be utilized with a ground-source heat pump, which takes advantage of the ground as an energy storage system [6,7]. In the energy piles system, the piles are used to absorb and transport thermal energy from the surrounding ground to buildings via fluid circulating in pipes placed within the piles. In fact, the thermal cycle can influence the loading of energy piles. Figure 2 displays the effect of thermal cycles of the energy piles system on pile stresses. Many studies have been carried out to investigate the geotechnical performance of piled foundations for ground-source heat-pump systems [8,9]. The main effects of temperature changes on pile behavior were assessed by the geotechnical numerical analysis method [10]. Applying thermal loads could induce a significant change in the stress–strain state of piles and the temperature change over the cross section of the piles should be designed to avoid high stress accumulated in piles [11,12]. Phase change materials are promising thermal energy shortage candidates that can be used to reduce the possible mismatch between thermal energy supply and demand. Applying phase change materials (PCMs) to piles can help in maintaining a stable temperature within the piles and can then influence the axial load acting on the piles. Among PCMs, paraffin is usually preferred as it is generally believed to be chemically inert, non-corrosive, show small volume changes during phase transition, innocuous, inexpensive, and recyclable. However, it has low thermal conductivity, which in turn, limits its application in thermal energy storage [13].

Expanded graphite (EG) and graphene nanoplatelets (GNPs) are safe, environmentally friendly, have low density and superior thermal conductivity. These are preferred over metal macro-scaled promoters, which have been used to increase PCMs thermal conductivity [14]. Sari and Karaipekli [15] prepared paraffin/expanded graphite composite PCM and found that composite PCM with 10 wt % of EG is a suitable thermal energy storage candidate. With this mass fraction of EG in composite, the thermal conductivity improved by approximately 273% in comparison to pure paraffin. Wang et al. [16] incorporated 90 wt % of polyethylene glycol into expanded graphite and found that the thermal conductivity of polyethylene glycol (0.2985 W·m^−1^·K^−1^) improved to 1.324 W·m^−1^·K^−1^. It was shown that the composite PCM is a promising candidate for latent heat storage applications. Xia et al. [17] showed that by incorporating 10 wt % of EG into paraffin, the composite PCM showed a 10-fold increase in thermal conductivity over pure paraffin. Mill et al. [18] improved the thermal conductivity of paraffin by two orders of magnitude using porous graphite matrices with paraffin. Zeng et al. [19] prepared Tetradecanol/EG composite PCM and showed that in comparison to pure tetradeconal (0.433 W·m^−1^·K^−1^), the thermal conductivity of the sample with 7 wt % of EG increased to 2.76 W·m^−1^·K^−1^. The optimum weight percentage of EG in Tetradecanol/EG composite PCM was suggested as 20 wt %.

In the recent past, graphene nanoplatelets (GNPs), which have high thermal conductivity and specific surface area, have been used and found to be suitable for PCM applications [20]. In order to enhance the composite PCM’s thermal conductivity, Mehrali et al. [21] used GNPs with different surface areas in palmitic acid (PA). The maximum percentage of PA absorbed by GNPs without any sign of leakage was found as 91.94 wt %. Moreover, GNPs with a surface area of 750 m^2^/g improved composite PCM’s thermal conductivity 10-fold with respect to pure palmitic acid. GNPs were used by Tang et al. [22] to improve the thermal conductivity of palmitic acid/high density polyethylene composite PCM. With 4 wt % of GNPs, the thermal conductivity was nearly 2.5 times higher than pure form-stable composite PCM. Silakhori et al. [20] showed that GNPs with 1.6 wt % improved the thermal conductivity of palmitic acid/polypyrole with an increase of 34.3%.

In this research, two kinds of carbon-based composite PCMs (expanded graphite-based PCM and graphite nanoplatelet-based PCM) were prepared for potential application in energy piles. Thereafter, a systematic study was performed and different characterization tests were carried out on two composite PCMs.

## 2. Materials and Methods

### 2.1. Materials

A technical grade paraffin, which is generally believed to be chemically inert, non-corrosive, show small volume changes during phase transition, innocuous and inexpensive, was used for this research [23]. As far as a carrier for PCM is concerned, expanded graphite (supplied by Qingdao Teng Sheng Da Carbon Machinery Co., Ltd., Qingdao, China) with an expansion ratio of 300 and technical-grade graphene nanoplatelets (supplied by Chinese Academy of Sciences Chengdu Organic Chemical Co., Ltd., Chengdu, China) were used. The properties of EG and GNPs are enlisted in Table 1.

### 2.2. Preparation of CPCMs

In this research, vacuum impregnation was used to prepare two kinds of composite phase change materials (CPCMs), i.e., expanded graphite-based PCM (EG–paraffin) and graphite nanoplatelet-based PCM (GNP–paraffin). The procedure adopted is as follows. At first, 100 g of supporting material (EG or GNPs) and 300 g paraffin were mixed together and put into a vacuum chamber for about 2 h to evacuate the air from the composite PCM. Thereafter, the vacuumed composite PCM was kept on the filter paper to remove the redundant paraffin. Finally, the percentage of PCM retained by GNPs was determined after removing the redundant PCM from the composite and keeping the PCM in an oven at 80 °C for three days. Moreover, during this period, the high absorption cushion paper was changed eight times on average to remove the extra PCM until the composite PCM mass became constant. The maximum amount of paraffin retained by EG and GNPs was 92.3% and 31.5% respectively.

### 2.3. Characterization Tests for CPCMs

#### 2.3.1. Micromorphology of CPCMs

The micromorphology of EG, GNPs and the CPCMs was examined using ESEM (Quanta 250 FEG, FEI Company, Hillsboro, OR, USA). The machine was operated under low vacuum in secondary-electron detection mode at an accelerating voltage of 15 kV. In order to obtain representative images, several regions of the powdered samples were observed. The SEM micrographs (Quanta 250 FEG, FEI Company, Hillsboro, OR, USA) were also captured for thermal energy storage cement paste while the energy dispersive spectrometer (EDS) was used to evaluate CPCMs dispersion in the cement paste.

#### 2.3.2. Chemical Compatibility of CPCMs

A FT-IR spectrometer was used to evaluate the chemical compatibility between the components of the CPCMs (Nicolet 6700; Thermo Electron Scientific Instruments Corp., Waltham, MA, USA). After mixing CPCM and KBr in a 1:30 (powder: KBr) ratio, the sample was pressed in “Manual Hydraulic Presess” at 10 ton for 1 min. Finally, the infrared spectrum was obtained by keeping the KBr pellets in the sample compartment. The scanning parameters were frequency ranging from 4000 to 400 cm^−1^ with a resolution of 4 cm^−1^.

#### 2.3.3. Thermal Capacity of the CPCMs

Thermal capacity of the CPCMs (DSC-Q200, TA Instruments Corp., Newcastle, PA, USA) was determined by using DSC. The sample was tested under nitrogen atmosphere in a temperature range of 0–60 °C at 2 °C/min heating/cooling rate at a flow rate of 40 mL/min. The results were extracted by using TA Instruments Universal Analysis software.

#### 2.3.4. Thermal Stability of CPCMs

The thermal stability of the CPCMs was evaluated by using TGA Q50 (TA Instruments Corp., Newcastle, PA, USA). The sample was tested under nitrogen atmosphere from room temperature to 600 °C with a heating rate of 20 °C/min and flow rate of 20 mL/min.

#### 2.3.5. Thermal Reliability of CPCMs

The thermal reliability of the CPCMs was determined with respect to change in thermal properties after 100 heating/cooling cycles. For this purpose, the sample was placed in the Temperature and Humidity programmable chamber manufactured by Dongguang Bell. The sample was subjected to a heating/cooling rate of 4 °C/min from 10 °C to 50 °C. Moreover, the temperature was maintained for 30 min at 50 °C and 10 °C, respectively. After thermal cycling, the FT-IR and DSC analyses were performed.

#### 2.3.6. Compressive Strength of Cement Paste Composite Containing CPCMs

The compressive strength of paste (20 × 20 × 20 mm^3^) incorporated with 0% and 10% CPCMs (EG- and GNP-based PCM) by weight of cement was evaluated at 28 days by applying a loading rate of 50 ± 10 N/s. The experimental matrix is given in Table 2. As far as the mixing of the ingredients is concerned, initially, the cement and CPCM were dry mixed and then a mixture of water and superplasticizer was added to the dry mixture.

## 3. Results and Discussions

### 3.1. Macro- and Micro-Morphology of Composite PCM

**Figure 3:**
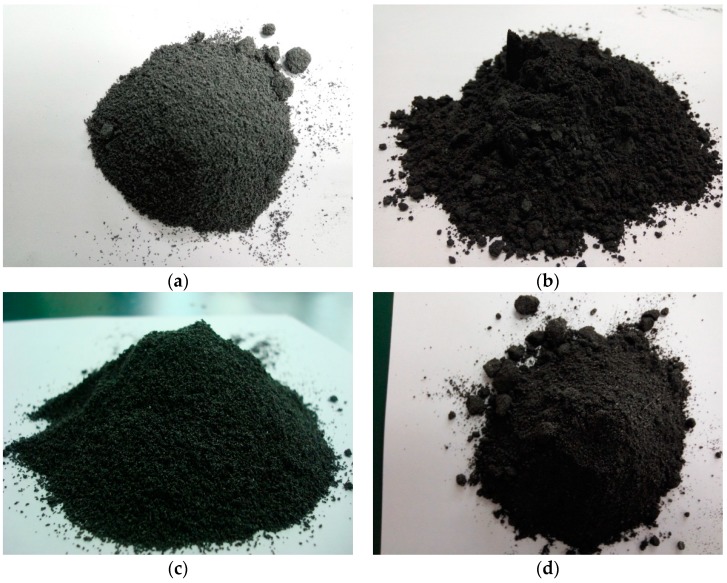
Appearance of the used materials in this study. (**a**) Expanded graphite (EG); (**b**) Graphene nanoplatelets (GNPs); (**c**) EG–paraffin CPCM; (**d**) GNPs–paraffin CPCM.

**Figure 4:**
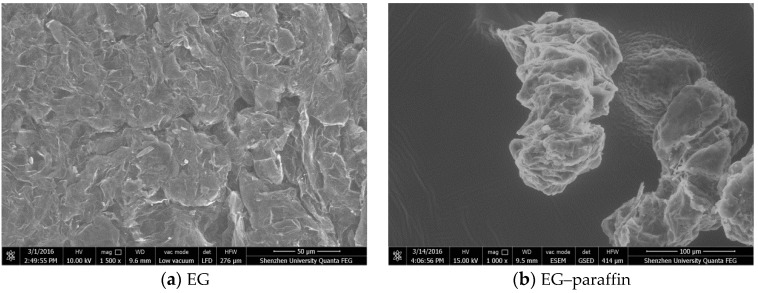

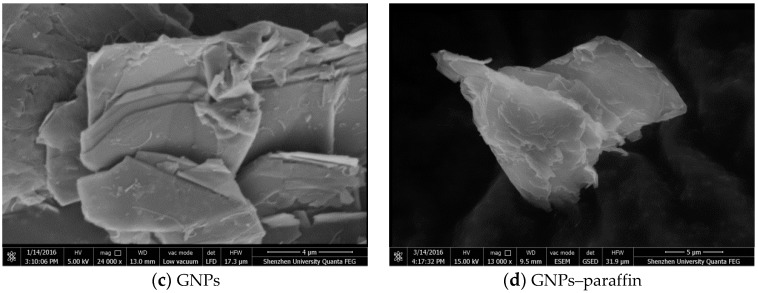
SEM micrographs of (**a**) Expanded graphite (EG); (**b**) EG–paraffin CPCM; (**c**) Graphene nanoplatelets (GNPs); (**d**) GNPs–paraffin CPCM.

The morphologies (at macro- and micro-scale) of EG, GNPs, EG–paraffin and GNP–paraffin are shown in Figure 3. In comparison to the light grey colour of EG powder (Figure 3a), the EG–paraffin sample showed a dark grey colour, which was due to the penetration of PCM in the liquid state. The micrograph of EG is shown in Figure 4. It is known from literature [24] that at micro-scale, EG shows a flattened irregular honeycomb network. The micrograph of EG–paraffin was similar to EG except that PCM showed the honeycomb structure due to the effect of capillary and surface tension forces. At macro level, the GNP powder showed black colour, which became darker due to the penetration of paraffin in the liquid state (Figure 3c,d). At micro-scale (Figure 4c), GNP particles (flaky in nature) showed smooth planer structure with a large surface area, which led to mechanically interconnected composites [20]. When the PCM was composed with GNP particles (Figure 4d), they have the tendency to absorb organic materials on their surface [20].

### 3.2. Chemical Compatibility of the CPCMs

**Figure 5:**
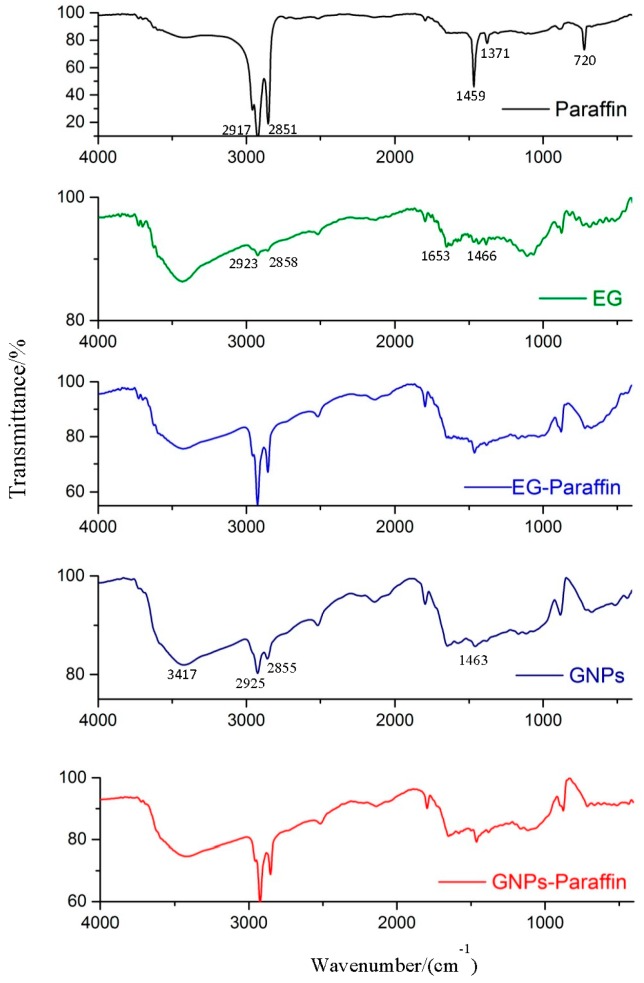
FT-IR spectra of paraffin, EG, GNPs, EG–paraffin, GNP–paraffin.

The FT-IR spectra of paraffin, expanded graphite, GNPs and composite PCMs are shown in Figure 5. The paraffin spectrum shows peaks at 2917 cm^−1^, 2851 cm^−1^, 1459 cm^−1^, 1371 cm^−1^ and 720 cm^−1^. The peaks at 2927 cm^−1^ and 2851 cm^−1^ are related to C–H stretching vibration of the methylene group [25,26,27], while the peak at 720 cm^−1^ is associated to the rocking vibration of the methylene group [25,28,29]. A strong peak (1597 cm^−1^) related to the C–H bending vibration of the methylene/methyl group [25] and a weak peak (1378 cm^−1^) corresponding to the C–H bending vibration of the methyl group can also be observed [25,28,29]. Finally, the shoulder in the region near 3430 cm^−1^ is linked to the OH stretching of the hydroxyl group [30].

The spectrum of EG shows a wider band at 3421 cm^−1^, which is linked to the stretching vibration of the OH group [31]. It also shows bands at 2923 cm^−1^ and 2858 cm^−1^ (symmetric and asymmetric stretching vibration of –CH_2_), 1653 cm^−1^ (–C=C– stretch structural vibration), and 1466 cm^−1^ (–C–C– stretch) respectively.

For GNPs, the wider peak at 3417 cm^−1^ is linked to the stretching vibration of the OH group [21] while the bands at 2925 cm^−1^, 2855 cm^−1^, and 1463 cm^−1^ are related to the symmetric and asymmetric stretching vibration of –CH_2_ and –C–C– stretch respectively.

The FT-IR spectra of EG–paraffin and GNP–paraffin CPCMs clearly depict that interactions are physical in nature. Therefore, the developed CPCMs are chemically compatible.

### 3.3. Thermal Properties of CPCMs

**Figure 6:**
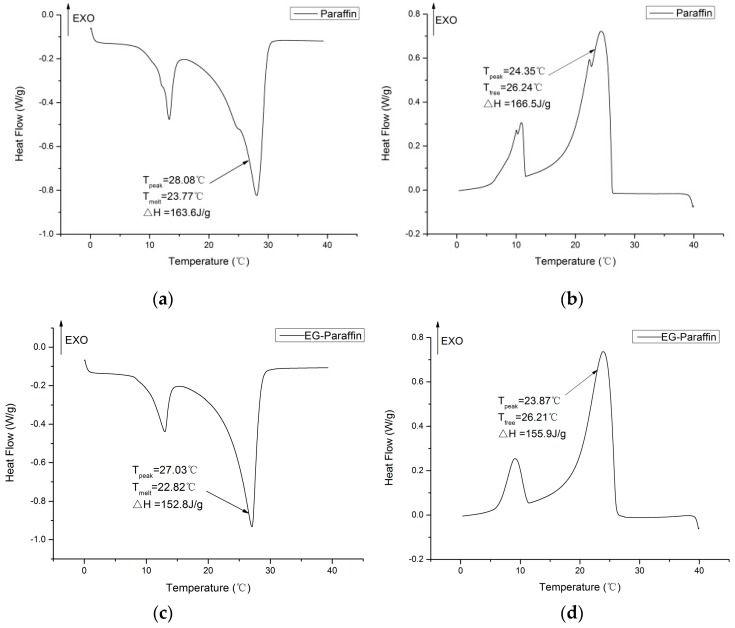

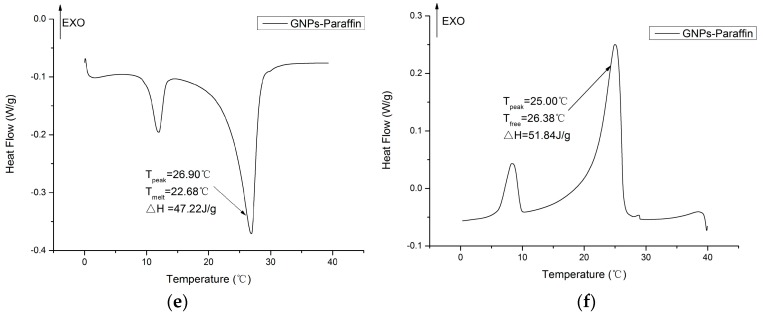
DSC thermograms—paraffin and CPCMs. (**a**) endothermic curve of paraffin; (**b**) exothermal curve of paraffin; (**c**) endothermic curve of EG-paraffin; (**d**) exothermal curve of EG-paraffin; (**e**) endothermic curve of GNPs-paraffin; (**f**) exothermal curve of GNPs-paraffin.

DSC was used to determine the thermal properties of paraffin, EG–paraffin and GNP–paraffin composites. The DSC curves are shown in Figure 6. The samples show two characteristic transition peaks, in which the minor peak represents the solid–solid phase change of paraffin while the major peak represents the solid–liquid phase change of paraffin [32]. The melting and freezing temperatures for paraffin were 23.77 °C and 26.24 °C while these temperatures were 22.88 °C and 26.21 °C for EG–paraffin and 22.68 °C and 26.88 °C for GNP–paraffin respectively. This shows that with the incorporation of EG and GNPs, the melting point of PCM decreased. The decrease in the melting temperature is believed to be due to the increase in heat transfer caused by the addition of EG and GNPs, which have higher thermal conductivities. It can also be observed that the difference in the peak melting and freezing temperatures is reduced by the incorporation of EG and GNPs in paraffin. For example, the peak temperature difference between the melting and freezing temperature of paraffin is 3.73 °C, while these values are 3.16 °C and 1.9 °C for EG–paraffin and GNP–paraffin composites respectively. For the PCM composites, the decrease in the peak temperature difference is believed to be due to the improved thermal conductivity of EG and GNPs. Moreover, GNP–paraffin composite has a greater capacity in reducing the temperature gap between the melting and freezing stage.

**Figure 7:**
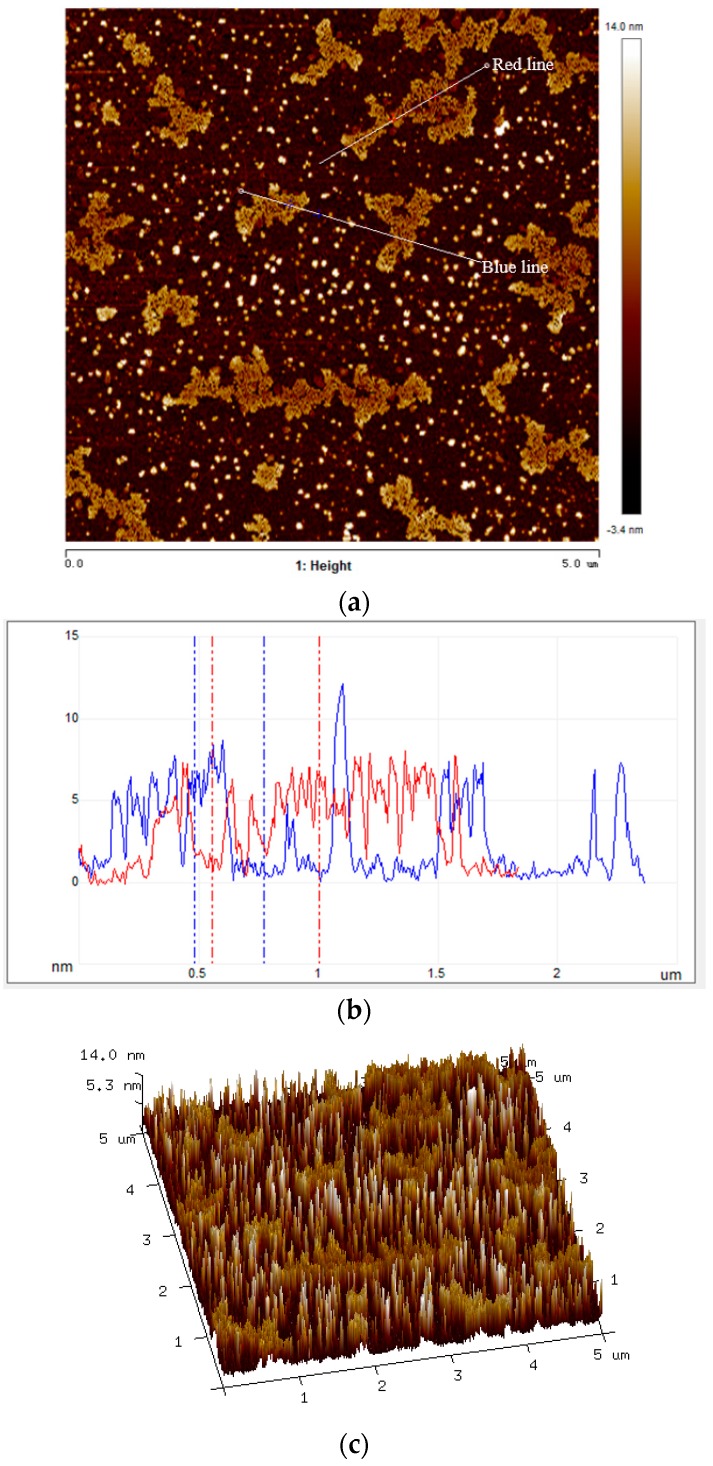
Tapping mode image of atomic force microscopy (AFM). (**a**) AFM Image of graphene (scan size: 5 × 5 µm); (**b**) Cross sections of two different directions; (**c**) Surface morphology of graphene by AFM 3D image.

In order to evaluate the better performance of GNP–paraffin composite, the morphology characteristics of GNPs, which can obviously influence the thermal conductivity were determined. Moreover, it is known that uniform thickness can help to ensure even distribution of the thermal conductivity for GNP–paraffin composite. Hence, atomic force microscopy (AFM) was employed to map the topographical and structural properties of graphene by investigating how well an AFM probe tip sticks to or is repelled by a surface, or how easy it is to press the probe tip into the surface. The AFM images of GNPs are shown in Figure 7. From Figure 7a,b, it can be seen that the thickness of most parts of GNPs along two sections with different directions (red line and blue line) ranges from 5 nm to 8 nm (Figure 7b). The surface morphology of GNPs in 3D (Figure 7c) shows that GNPs used in this research have an even surface, which means that in terms of thermal conductivity, the quality of GNPs is good. Hence, for this reason, the efficiency of GNP–paraffin (in terms of thermal conductivity) is superior in cement-based materials than in EG–paraffin. Although there are some defects in the GNPs, proper defects in GNPs can contribute to providing good composition between GNPs and PCMs.

As far as the latent heats of fusion and solidification are concerned, they were found to be 163.6 J/g and 166.5 J/g for paraffin, 152.8 J/g and 155.9 J/g for EG–paraffin and 51.84 J/g and 47.22 J/g for GNP–paraffin. The encapsulation efficiency determined by Equation 1 was found to be 93.51% for EG–paraffin and 30.02% for GNP–paraffin respectively.

η(%) = (ΔH_m,EG-PCM/GNP-PCM_ + ΔH_f,EG-PCM/GNP-PCM_)/(ΔH_m,PCM_ + ΔH_f,PCM_) × 100% (1)

In research conducted by Mehrali et al. [21], in which they used GNPs with surface areas of 300, 500 and 750 m^2^/g, the maximum percentage of palmitic acid retained by GNPs was found to be 77.99%, 83.1% and 91.94% respectively. In comparison, for the GNPs used in this research (surface area of 100 m^2^/g) 30 wt % retained PCM is acceptable. When the cost of GNPs with different surface areas is compared, GNPs with 750 m^2^/g cost 150 USD/g while 100 m^2^/g cost only 0.2 USD/g. This shows that the GNPs used in this research with a surface area of 100 m^2^/g are 750 times cheaper than GNPs with a surface area of 750 m^2^/g. Some other reasons for the difference in the thermal energy storage capacity of GNP composites are as follows. In research conducted by Mehrali et al. [21], they used palmitic acid, which has lower viscosity than paraffin. It is believed that PCM with lower viscosity was retained well by the GNPs. Secondly, the researchers used a hydraulic press to compact the PCM composite, which might have allowed the extra PCM to stay with the GNPs. However, in our case, the percentage of PCM retained by the GNPs was determined after removing the redundant PCM from the composite, by keeping the PCM in an oven at 80 °C for three days. Moreover, during this period, the high absorption cushion paper was changed eight times on average to remove the extra PCM until the composite PCM mass became constant.

The values of latent heat thermal energy storage obtained from this research were compared with those available in literature (Table 3). The results depicted in Table 3 are promising and therefore the developed composite PCMs are potential thermal energy storage candidates for energy piles and buildings.

### 3.4. Thermal Stability of the CPCMs

**Figure 8:**
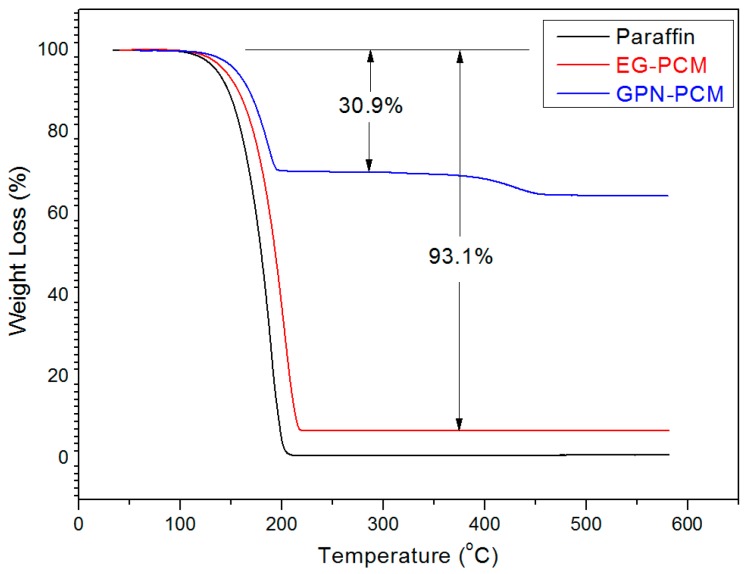
TGA thermograms of the paraffin and CPCMs.

The thermal stability of composite PCM was determined to ensure that it is stable in the working temperature range. The TGA thermograms of pure paraffin and composite PCMs are shown in Figure 8. It can be seen that the initial decomposition temperature of composite PCMs shifted to a higher temperature when compared to pure paraffin, indicating an increase in the thermal stability of composite PCM. The results also indicate that EG and GPN were advantageous in slowing down the degradation process. It is believed that the thermal energy is initially absorbed by EG and GPN; hence, enough energy to initiate paraffin decomposition only becomes available at a slightly higher temperature [48,49]. It is also suggested that the surfaces of EG and GPN might have adsorbed the volatile decomposition products which in turn retarded its diffusion out of the sample and hence the mass loss was only observed at a slightly higher temperature [48,49]. Finally, the observed weight loss of paraffin in composites is in line with the vacuum impregnation results, indicating homogeneous preparation of composite PCM. Conclusively, composite PCMs are thermally stable in the working temperature range.

### 3.5. Thermal Reliability of CPCMs

**Figure 9:**
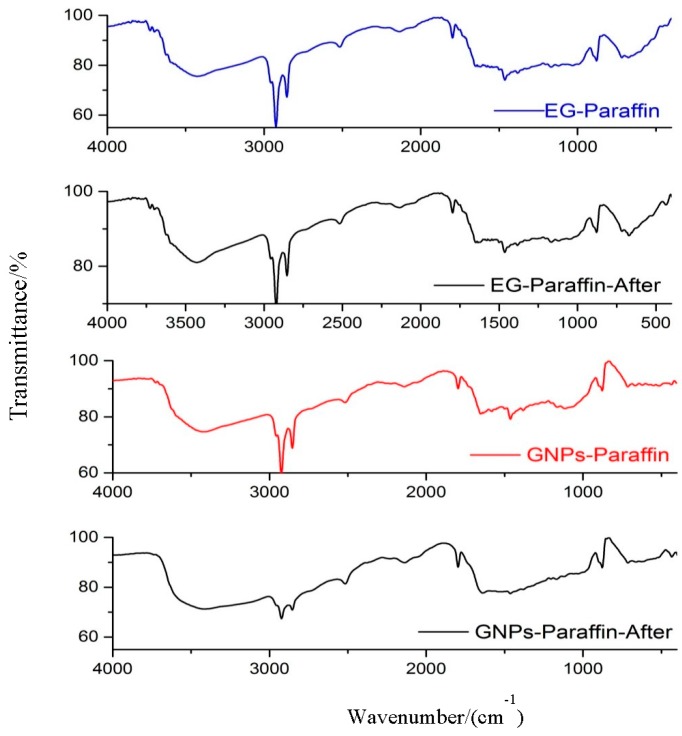
FT-IR of composite PCMs before and after thermal cycling.

The developed composite PCMs were subjected to 100 thermal cycles, and FT-IR and DSC were used to determine the changes in chemical structure and thermal properties. The FT-IR spectra of the developed composite PCM are shown in Figure 9. The finger print of the composite PCM (before and after thermal cycling) shows no obvious difference, clearly suggesting that thermal cycling did affect the chemical structure of the developed composite.

**Figure 10:**
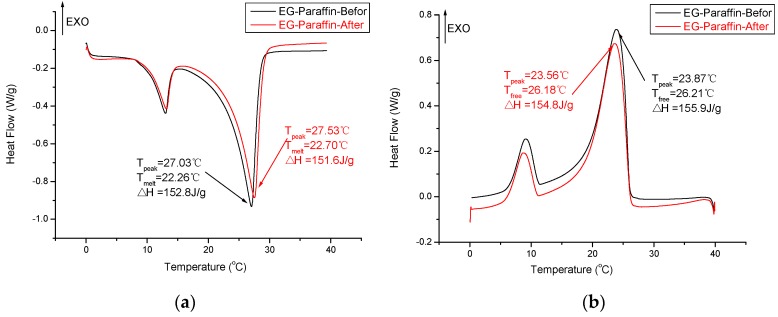

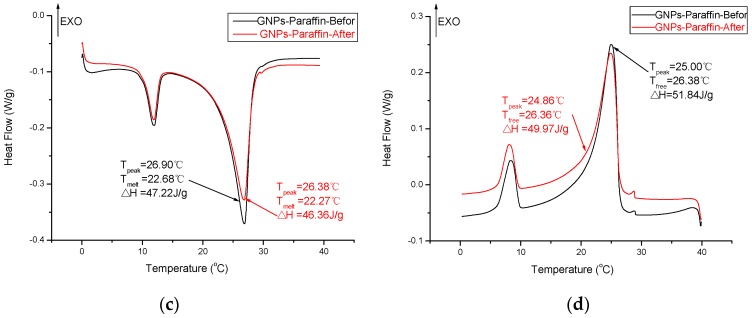
DSC thermograms of composite PCM before and after 100 thermal cycles. (**a**) endothermic curve of EG-paraffin; (**b**) exothermal curve of EG-paraffin; (**c**) endothermic curve of GNPs-paraffin; (**d**) exothermal curve of GNPs-paraffin.

The thermal properties of composite PCM (EG–paraffin and GNP–paraffin) before and after thermal cycling are shown in Figure 10. After thermal cycling, the melting and freezing temperatures for EG–paraffin changed by −0.44 °C and 0.03 °C respectively, while the latent heat of melting and freezing changed by 1.2 J/g and 1.1 J/g respectively. For GNP–paraffin, the melting and freezing temperatures changed by 0.41 °C and 0.02 °C respectively, while the latent heat of melting and freezing changed by 0.86 J/g and 1.87 J/g respectively. This shows that the changes observed in phase change temperature and latent heat storage capacity are smaller and therefore the developed composite PCMs are thermally reliable and suitable for thermal energy storage applications.

### 3.6. Compressive Strength of Cement Paste Containing CPCMs

**Figure 11:**
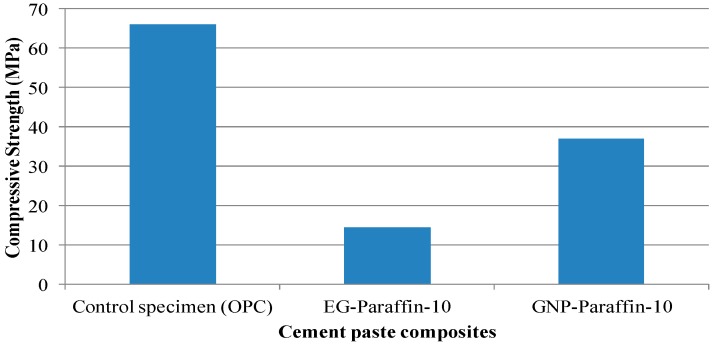
Compressive strength of cement paste incorporated with 0 and 10 wt % CPCMs (EG and GNP-based PCM).

The compressive strength results of cement paste incorporated with 0 and 10% CPCMs (EG- and GNP-based PCM) by weight of cement are presented in Figure 11. The compressive strength of cement paste containing 10 wt % EG-PCM and GNP-PCM was found to be 14.6 MPa and 37 MPa respectively. The percentage reduction in compressive strength for these mixes was 77.9% and 44% respectively. In research conducted by Zhang et al. [24], the decrease in percentage of cement mortar with 2.5% *n*-octadecane/EG composite PCM was found to be 55%. We would like to mention here that the compressive strength of GNP-PCM cement paste was 37 MPa and is acceptable for many applications as mentioned in literature [24,50,51,52] and Chinese National standard (GB 50574-2010) for building materials.

## 4. Conclusions

In this research, two kinds of composite PCM i.e., expanded graphite-based PCM and graphite nanoplatelet-based PCM, were prepared using vacuum impregnation. The conclusions drawn are as follows.

(1) The maximum percentage of paraffin retained by EG and GNPs through vacuum impregnation was 92.3% and 31.5% respectively. This data is similar to the data obtained from TGA results (93.1% and 30.9%), thus testifying the homogenous preparation of composite PCM. From micro-morphology, it was found that PCM possessed a honeycomb structure of EG due to capillary and surface tension forces while for GNPs, the larger surface area provided favourable conditions to absorb PCM.
(2) From the FT-IR analysis, the interaction between components of composite PCM was physical in nature and therefore the components of prepared carbon-based composites are chemically compatible with each other.
(3) The DSC analysis showed that the developed carbon-based PCMs possess considerable latent heat and can therefore be a potential candidate for energy piles.
(4) From thermal stability results, it was found that the incorporation of carbon-based materials in PCM shifted the initial decomposition to a higher temperature, indicating an increase in the thermal stability of composite PCM. Furthermore, the developed composite PCM did not show any sign of degradation below 100 °C. Hence, it is thermally stable and can be utilized for thermal energy storage applications.
(5) The chemical structure and thermal properties of developed carbon-based composite PCMs were not affected by thermal cycling. Therefore, the composite PCM is thermally reliable and can be used for latent heat storage applications.
(6) The compressive strength of cement paste containing 10 wt % GNP-PCM was found to be 37 MPa and it therefore has potential application for structural purposes.

## Figures and Tables

**Table 1:** Properties of expanded graphite (EG) and graphene nanoplatelets (GNPs).

Type	Particle Size	Diameter	Expanded Time	Nanosheet Number
Expanded graphite	~0.5 mm	/	300	/
Graphene	/	5–7 μm	/	<20

**Table 2:** Mix proportion (mass ratio) of composite phase change materials (CPCM) in cement paste.

Cement Paste	Cement	Water	CPCMs	Superplasticizer (wt %)
Control specimen (OPC)	1	0.35	0	0.15
EG–paraffin-10	1	0.35	0.1	0.3
GNP–paraffin-10	1	0.35	0.1	0.3

**Table 3:** Thermal properties comparison of composite PCMs with phase change temperature in the human comfort zone.

Composite PCMs	Melting Point (°C)	Latent Heat (J/g)	Reference
Dodecanol (25–30 wt %)/gypsum	20	17	[33]
Capric-myristic acid (20 wt %)/Vermicuilite	19.8	27	[34]
Capric-lauric acid + fire retardant (25–30 wt %)/gypsum	17	28	[35]
Butyl stearate (25–30 wt %)/gypsum	18	30	[35]
Emersest2326/gypsum	16.9	35	[36]
Capric-lauric acid (26 wt %)/gypsum	19	35.2	[37]
Capric-myristic acid (25 wt %)/gypsum	21.1	36.2	[38]
Erythritol tetrapalmitate ester (18 wt %)/ cement	21.9	37.2	[39]
Capric-lauric acid (26 wt %)/gypsum	18.49	39.13	[40]
Propyl palmitate (25–30 wt %)/gypsum	19	40	[33]
Capric-palmitic acid (25 wt %)/gypsum	22.9	42.5	[41]
Paraffin/GNPs	22.68	47.22	This study
Decanoic/Dodecanoic acid/Diatomite	16.7	66.8	[42]
RT20 (58%)/Montmorillonite	23	79.3	[43]
PCM-clay composite (PMMT1-4)	16–17	82–128	[44]
Paraffin/Diatomite/CNTs	27.12	89.40	[45]
Capric acid (55%)/Expanded perlite	31.8	98.1	[46]
Octadecane (70%)/Expanded graphite	29.6	138.8	[47]
Paraffin/Expanded graphite	22.82	152.8	This study

## Abbreviations

PCM	Phase change materials
CPCM	Composite phase change material
EG	Expanded graphite
GNP	Graphene nanoplatelet
EG-PCM	Expanded graphite-based PCM
GNP-PCM	Graphene nanoplatelets-based PCM
